# Routine Fecal Occult Blood Screening and Colorectal Cancer Mortality in Sweden

**DOI:** 10.1001/jamanetworkopen.2024.0516

**Published:** 2024-02-27

**Authors:** Johannes Blom, Deborah Saraste, Sven Törnberg, Håkan Jonsson

**Affiliations:** 1 Department of Surgery, Södersjukhuset, Stockholm, Sweden; 2 Department of Clinical Science and Education, Karolinska Institutet, Stockholm, Sweden; 3 Department of Oncology-Pathology, Karolinska Institutet, Stockholm, Sweden; 4 Department of Epidemiology and Global Health, Umeå University, Umeå, Sweden

## Abstract

**Importance:**

Population-based colorectal cancer (CRC) screening programs are implemented worldwide, but there are difficulties evaluating their effectiveness. The magnitude of routine CRC screening effectiveness regarding cancer-specific mortality is unclear.

**Objective:**

To evaluate cancer-specific mortality associated with early vs late or no invitation for routine CRC screening using fecal occult blood testing.

**Design, Setting, and Participants:**

This prospective cohort study was performed in the region of Stockholm-Gotland, Sweden, between January 1, 2008, and December 31, 2021. All individuals of the target population of screening born from 1938 to 1954 were included. Data were analyzed from December 12, 2022, to June 25, 2023.

**Exposures:**

Individuals were invited early (2008-2012), late (2013-2015), or not at all to screening with biennial guaiac-based fecal occult blood test. The early invitation group was considered the exposure group and the late or no invitation group was considered the control group.

**Main Outcomes and Measures:**

The main outcome was cancer-specific mortality, defined as CRC registered in the Cancer Register with CRC as underlying cause of death in the Cause of Death Register. Excess mortality was calculated as all-cause deaths among the individuals with CRC subtracted from the expected number of deaths had they not had CRC. Poisson regression analysis based on deaths and person-years was used to estimated mortality rate ratio (RR) with 95% CIs, adjusted for follow-up years and attained age.

**Results:**

In total, 379 448 individuals (193 436 [51.0%] female) were invited for CRC screening, including 203 670 individuals in the exposure group and 175 778 in the control group. The mean screening participation rate was 63.3%, and there was a maximum of 14 years follow-up. There were 834 CRC deaths in 2 190 589 person-years in the exposure group, compared with 889 CRC deaths in 2 249 939 person-years in the control group. Individuals who underwent early CRC screening had reduced adjusted risk of CRC mortality (RR, 0.86; 95% CI, 0.78-0.95) and excess mortality (RR, 0.84; 95% CI, 0.75-0.93).

**Conclusions and Relevance:**

This prospective cohort study of routine screening with fecal occult blood testing found a 14% decrease in CRC mortality associated with screening. The true association of screening with reduced mortality is expected to be higher due to some coexistence of testing in the control group and CRC deaths diagnosed more than 2 years after screening.

## Key Points

**Question:**

Is routine screening with fecal occult blood test (FOBT) associated with reduced colorectal cancer (CRC) mortality?

**Findings:**

In this cohort study of 379 448 individuals, there was a statistically significant 14% decreased CRC mortality among individuals who received early invitation to FOBT screening compared with individuals who received late or no invitation after a maximum of 14 years of follow-up.

**Meaning:**

These findings suggest that population-based CRC screening with FOBT was associated with decreased CRC mortality.

## Teaser

This population-based cohort study assesses rates of colorectal cancer–specific mortality among individuals invited for early vs late or no screening using fecal occult blood testing.

## Introduction

Secondary prevention of colorectal cancer (CRC) with screening has the potential to reduce deaths from the disease by detecting the cancer at an early, curable stage. Larger precursors, such as adenomatous polyps (adenomas), and cancer may bleed, enabling the measurement of invisible (occult) blood in the stool with fecal occult blood tests (FOBT) before disease symptoms occur.

In 2003, the European Commission issued recommendations to screen for CRC with FOBT in men and women aged 50 to 74 years,^1^ and in 2006, national CRC screening programs started in some European countries, such as England and Italy.^2,3^ The recommendations were based on results from randomized screening trials assessing guaiac-based FOBT (gFOBT) and reporting a CRC mortality reduction of approximately 15%.^4,5,6^ In 2010, European Guidelines for Quality Assurance in CRC screening were issued,^7^ and in 2022, fecal immunochemical testing (FIT) was recommended.^8^ FIT is a FOBT with higher sensitivity and adherence with invitation compared with gFOBT^9,10^ and is expected to be at least as effective.^11^ Still, there is a lack of observational studies evaluating the association of CRC mortality with routine screening programs using FOBT.

In 2008, the region of Stockholm-Gotland, encompassing approximately 25% of the Swedish population, initiated a CRC screening program.^12^ Influenced by experiences from Finland,^2,13^ the program gradually invited men and women aged 60 to 69 years to undergo biennial gFOBT. If results were positive, a referral for colonoscopy was undertaken. In 2015, gFOBT was replaced with FIT.^10^ The aim of this study was to evaluate screening program effectiveness on CRC mortality by comparing birth cohorts invited early to screening with those not invited or invited late.

## Methods

This cohort study was approved by the Ethics Review Board of Sweden and in accordance with the Declaration of Helsinki. Informed consent was waived because all data were deidentified. This study is reported following the Strengthening the Reporting of Observational Studies in Epidemiology (STROBE) reporting guideline.

### The Screening Program

The screening program was centrally organized at the Stockholm-Gotland Regional Cancer Centre (RCC), Sweden, which was responsible for sending invitations, registration, and reporting outcomes of all parts of the screening process and ensuring quality control. Of 20 regional endoscopy clinics, 5 were contracted to perform all assessment colonoscopies. There were no other CRC screening activities in the region. The target group of screening comprised all residents in the region aged 60 to 69 years according to the Swedish population register, without any exclusion. In Sweden, all residents have a unique national registration number used in all population statistics, including health care.

An invitation to screening was sent from RCC to the home of the individuals, without involvement of primary health care physicians.^12^ The invitation included information on CRC screening, a test kit with 3 gFOBT test cards (Hemoccult-test; Beckman Coulter), instructions on how to take the test, and a prepaid return envelope. The test was not rehydrated before analysis and has demonstrated a screening program sensitivity of approximately 22% to 52%, although lower in women than in men.^14^ Participants with test results negative for occult blood (ie, 3 negative test cards) were informed by letter and, in case of false-negative test, advised to seek a physician if they had bowel symptoms. The individuals with test results positive for occult blood (ie, positive results on ≥1 of 2 panels of the 3 test cards) were electronically referred to the contracted endoscopy clinic that was responsible for assessment of individuals residing in the catchment area and that provided a colonoscopy within 2 weeks. Participants with invalid tests were sent new test kits, and invitees with no test results registered at the laboratory were sent a reminder after 8 weeks. Due to a higher level of interval cancers among women,^14^ the program changed from gFOBT to FIT (OC-Sensor; Eiken Chemical) in September 2015, with a cutoff level of 40 μg Hb/g feces for a positive result in women and 80 μg Hb/g for a positive result in men.

The invitations were sent biennially for 10 years, ie, 5 screening rounds, following the same procedure. Patients with cancer diagnosed at a screening episode were referred by the endoscopy clinic to surgery, while patients with advanced adenomas removed at screening colonoscopy, ie, adenomas of 10 mm or greater with high-grade dysplasia or villous histology, serrated polyps with dysplasia or 10 mm or greater in size or 3 or more low-risk adenomas, were referred to an adenoma surveillance program.

### Study Cohort

The study was performed on a cohort of 392 190 individuals who resided in the region of Stockholm-Gotland, Sweden, in 2008 to 2012 and were born between 1938 and 1954. In 2007, the program randomized birth years to determine the starting year for screening invitations, with 2008 as the initial year and 2015 as the final year for inviting individuals to commence screening. The number of screening invitations per individual varied due to age at first invitation, with 5 as maximum (ie, 10 years). There were also birth cohorts never invited due to allocation to the no invitation group. Some birth cohorts were allocated to early start, ie, invitation to screening during 2008 to 2012 (exposure group), while the other birth cohorts never got an invitation or had their first invitation in 2013 to 2015 (control group) (Table 1). There were no other systematic differences between groups than year of invitation.

**Table 1.** Evaluation Group, Planned Year of Start of Screening, Age at Screening Start, Start of Follow-Up, Total Number, and Proportion of Women by Birth Year

Birth year	Group^a^	Screening start	Age at start, y	Start of follow-up	Proportion women, %	Total No.
1938	Control	No screening	NA	2008	52.5	15 208
1939	Control	No screening	NA	2008	51.4	16 194
1940	Exposure	2009	69	2009	51.1	15 402
1941	Control	No screening	NA	2008	51.5	18 322
1942	Exposure	2008	66	2008	51.0	20 808
1943	Exposure	2011	68	2011	51.2	21 864
1944	Exposure	2009	65	2009	51.2	24 889
1945	Control	2013	68	2008	51.0	26 481
1946	Exposure	2008	62	2008	51.5	26 265
1947	Control	2013	66	2008	51.0	26 551
1948	Exposure	2012	64	2012	52.4	23 888
1949	Exposure	2009	60	2009	51.5	24 043
1950	Exposure	2010	60	2010	50.3	23 613
1951	Control	2013	62	2008	50.7	23 729
1952	Exposure	2012	60	2012	50.0	22 898
1953	Control	2015	60	2008	49.8	24 660
1954	Control	2014	60	2008	49.3	24 633

Abbreviation: NA, not applicable.

^a^ Exposure group: individuals invited to early start of screening (2008-2012). Control group: individuals invited to late start of screening (2013-2015) or were never invited.

### Data Retrieval

Data on the cohort individuals, including information on emigration, were retrieved from Statistics Sweden. By using the unique national registration number, the cohort data were then linked to the national cancer register and the cause of death register at the National Board of Health and Welfare to retrieve data on diagnoses of CRC from 1958 to 2020 and deaths and the underlying cause of death (UCD; in case of CRC) from 2008 to 2021.^15,16^ Data were further linked to the regional screening register at RCC Stockholm-Gotland to retrieve data on program performance, eg, dates of invitation, participation, and results of the tests and assessment colonoscopies.

### Diagnosis and Cause of Death

The Swedish national cancer register uses the *International Classification of Diseases, Revision 7* (*ICD-7*) with CRC diagnosis code 153.X (malignant neoplasm of large intestine, except rectum) or 154.0 (malignant neoplasm of rectum) excluding the morphology (histology) codes C24; 091 (neuroendocrine tumor), 093 (lymphoma), 094 (adenoma), 144 (squamous cell carcinoma), or 793 (gastrointestinal stroma tumor). Death attributed to CRC as the underlying cause was determined using the *International Statistical Classification of Diseases and Related Health Problems, Tenth Revision (ICD-10)*, specifically coded as C18.X–C20.X (malignant tumor in colon or rectum), within the cause of death register.

### Follow-Up

For the exposure group, the individual start of follow-up was defined as the date of the first invitation to the screening program, and for all individuals in the control group the start of follow-up was defined as January 1, 2008. All individuals were followed until possible emigration (the first emigration registered after start of follow-up), death, or December 31, 2021, whichever came first.

### Data Cleaning Process

**Figure 1.**
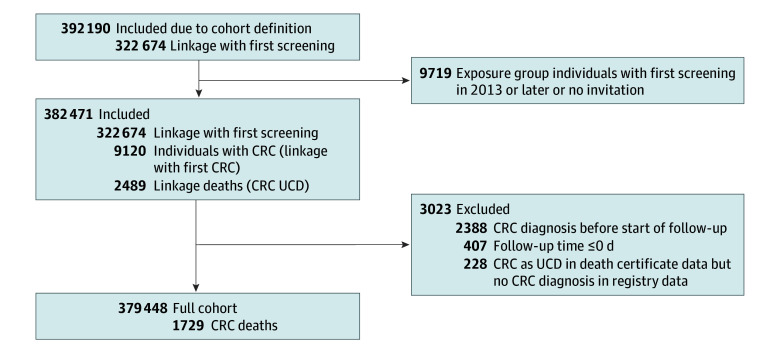
Cohort Selection Flowchart CRC indicates colorectal cancer; UCD, underlying cause of death.

Individuals in the exposure group never invited or with first screening later than January 1, 2013 were excluded. Furthermore, individuals with CRC diagnosis before the start of follow-up were excluded, since they could not be affected by the screening program. There were 228 individuals with death certificate–only data, ie, individuals with CRC listed as the UCD, but they had no diagnosis of CRC in the national cancer register. They were excluded from the primary analysis but incorporated into a sensitivity analysis. More details of the data cleaning process are provided in Figure 1.

### Statistical Analyses

The main end point of the study was death with CRC as UCD. The rate ratio (RR) of CRC mortality was calculated for the exposure group vs the control group. Since individuals with CRC diagnosis before the start of follow-up were removed, this is often called *incidence-based mortality*. We also determined the timing of the CRC diagnosis in relation to the most recent screening invitation for individuals who died from CRC in the exposure group.

The mortality was adjusted for follow-up year and attained age using Poisson regression. The follow-up was divided into cells with number of person-years and number of CRC deaths per follow-up year, year of attained age, and group. Poisson regression was performed with logarithm of the number of person-years as offset to estimate the mortality RR adjusted for follow-up year and attained age. The analysis was also extended by including sex.

Determining a singular UCD in individual deaths, especially when there is a known diagnosis, like CRC, can be challenging. Excess mortality offers an alternative approach for calculating the cause of death independently of individual specifics. We assume the risk of death for a patient with CRC to be the normal mortality rate plus an additional mortality attributed to the disease. Subtracting the normal mortality provides an estimate of CRC mortality. The numerator, representing the excess number of deaths, was calculated by subtracting the expected number of deaths individuals would have had without CRC from the all-cause deaths among the individuals with CRC. The expected number of deaths was calculated as the product of the person-years among individuals with CRC and the population’s all-cause mortality rate. The calculations were made by sex, age, and calendar year and then summarized. To determine excess mortality, the excess number of CRC deaths is then divided by person-years, following the standard mortality calculation approach. Excess mortality was analyzed in a similar manner to mortality based on the UCD, using Poisson regression. However, the Poisson analysis was adjusted due to the higher variance of the excess number of cases compared to its expected value; the distribution is not Poisson. The variance was adjusted by adding the expected number of deaths. We conducted a sensitivity analysis by including the 228 individuals with CRC as UCD on death certificates only but without diagnosis of CRC in the national cancer register.

We used R statistical software version 4.2.2 (R Project for Statistical Computing) for all calculations. Statistical significance was defined as a *P* < .05 for a 2-sided hypothesis. For RRs, this corresponds to a 95% CI not including 1.0. Data were analyzed from December 12, 2022, to June 25, 2023.

## Results

**Figure 2.**
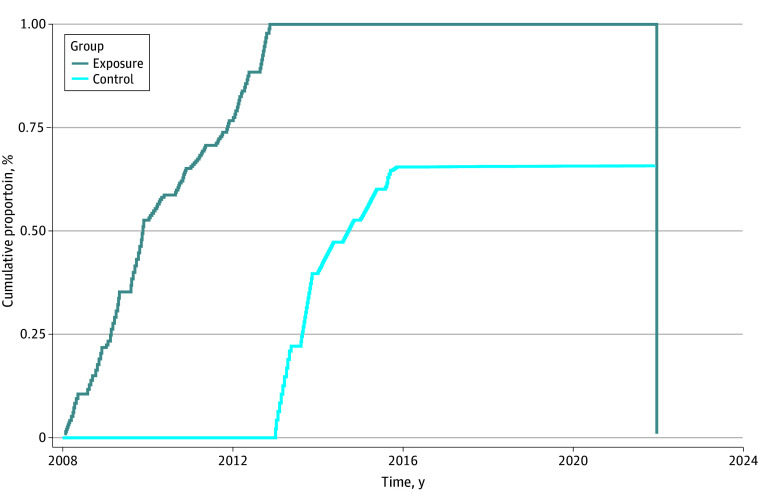
Cumulative Proportion of Invited to First Screening

After data cleaning, there were 379 448 individuals (193 436 [51.0%] female) in the cohort, including 203 670 individuals in the exposure group and 175 778 individuals in the control group. The maximum follow-up was 14 years, with a mean (SD) of 10.8 (2.7) years in the exposure group and 12.8 (3.0) years in the control group. In the control group, 60 162 individuals (34.2%) were never invited for screening, while 115 616 individuals (65.8%) were invited to participate in at least 1 screening round. The time to first screening per group is illustrated in Figure 2. The mean (SD) age at individual mid–follow-up was 68.9 (3.4) years for the exposure group 67.0 (5.6) years for the exposure group. Screening participation increased by individual round, with a mean of 63.3% and a pronounced increase from 2015, at the time of change from gFOBT to FIT (Table 2).^10^

**Table 2.** Invited and Participating Individuals per Test Method and Year

Year	Test method	Invited, No.	Participants, No. (%)
2008	gFOBT	44 486	28 189 (63.4)
2009	gFOBT	62 709	37 477 (59.8)
2010	gFOBT	67 902	40 838 (60.1)
2011	gFOBT	68 772	42 508 (61.8)
2012	gFOBT	93 676	53 227 (56.8)
2013	gFOBT	114 768	69 787 (60.8)
2014	gFOBT	112 640	63 814 (56.7)
2015	gFOBT^a^	82 202	48 221 (58.7)
2015	FIT	5744	4060 (70.7)
2016	FIT	85 936	59 067 (68.7)
2017	FIT	62 392	43 832 (70.3)
2018	FIT	61 754	43 760 (70.9)
2019	FIT	40 005	28 732 (71.8)
2020	FIT	40 174	29 209 (72.7)
2021	FIT	38 354	28 104 (73.3)
Total	NA	981 514	620 825 (63.3)
Never invited	NA	60 162	NA

Abbreviations: FIT, fecal immunochemical testing; gFOBT, guaiac-based fecal occult blood test; NA, not applicable.

^a^ The screening program changed from gFOBT to FIT September 1, 2015.

### Main Outcome

There were 834 CRC deaths in 2 190 589 person-years in the exposure group and 889 CRC deaths in 2 249 939 person-years in the control group (Table 3). Individuals who underwent early CRC screening had reduced risk of CRC mortality in unadjusted analysis (RR, 0.96; 95% CI, 0.88-1.06) and after adjustment for follow-up years and attained age (RR, 0.86; 95% CI, 0.78-0.95). The main result did not change when sex was included in the model. However, women had a significantly lower CRC mortality compared with men (RR, 0.67; 95% CI, 0.61-0.74). There was no significant interaction between group and sex (RR for screening for women vs men, 0.94; 95% CI, 0.78-1.14).

**Table 3.** Deaths With CRC as Underlying Cause of Death and Person-Years for the Total Cohort

Group^a^	All individuals, No.		Individuals with CRC, No.				Difference, No.
	Person-years	CRC deaths	Person-years	All-cause deaths	Expected deaths	Excess deaths	
Exposure	2 191 000	834	14 573	1160	269.2	890.8	56.8
Control	2 250 000	889	15 102	1283	307.6	975.4	86.4

Abbreviation: CRC, colorectal cancer.

^a^ The exposure group is individuals invited to early-start screening (2008-2012). The control group is individuals invited to late-start screening (2013-2015) or were never invited.

### Excess Mortality

There were 14 573 person-years and 1160 all-cause deaths among individuals with CRC in the exposure group and 15 102 person-years and 1283 all-cause deaths among individuals with CRC in the control group (Table 3). The resulting excess number of deaths was 890.8 in the exposure group and 975.4 in the control group, which is 6.8% higher than using the UCD in the exposure group and 9.7% higher than in the control group. Compared with the control group, the exposure group had reduced risk of excess mortality before adjustment (RR, 0.94; 95% CI, 0.86-1.03) and after adjustment for follow-up year and attained age (RR, 0.84; 95% CI, 0.75-0.93), ie, lower than the risk of CRC as UCD.

### CRC Diagnosis

Of 834 CRC deaths in the exposure group, 435 individuals (52.2%) received their CRC diagnosis more than 2 years after the last screening invitation. Of the remaining 399 CRC deaths, 204 individuals (51.1%) participated at the last screening round preceding the diagnosis.

### Sensitivity Analysis

A total of 109 individuals in the exposure group and 119 individuals in the control group with CRC as UCD in their death certificate data but no diagnosis in the national registry were included in a sensitivity analysis. The CRC mortality RR changed only marginally after adjustment for follow-up year and attained age (RR, 0.87; 95% CI, 0.79-0.96).

## Discussion

This cohort study found a 14% decrease in CRC mortality in the cohort of individuals aged 60 to 69 years who were invited to screening during the first 5 years of program implementation, compared with those not invited or with late-start invitations. The 14% mortality reduction is in line with the efficacy of 15% demonstrated by the randomized trials with biennial invitation to gFOBT screening (RR, 0.85; 95% CI 0.78-0.92),^17^ a result that has been difficult to render unbiased from observational studies of ongoing screening programs to date.

The demonstrated CRC mortality reduction was likely influenced by several factors. Some of the influencing factors could include the screening test, participation rate, adherence with follow-up colonoscopy, organization and administration, demands on the contracted units involved in the whole screening process, and accurate and complete monitoring of all data from invitation to the cause of death register.

Randomization of birth-cohorts to implement population-based CRC screening with gFOBT was first adopted in Finland in 2004, inviting men and women aged 60 to 69 years biennially, with the noninvited controls gradually screened after an implementation period of 6 years.^18^ At the first evaluation of program effectiveness, the exposure group and control group were approximately the same size as in this study (180 000 in each group), but no difference was observed regarding CRC mortality (RR, 1.04; 95% CI, 0.84-1.28]),^19^ probably due to the short median follow-up of 4.5 years.

Observations from the Dutch FIT-screening program of individuals aged 55 to 75 years, implemented during 2014 to 2019, also did not observe a difference in CRC mortality after the program introduction.^20^ This could be due to the shorter follow-up, but also a dilution of the end point, since mortality was not analyzed related to invitation to the program. However, the Italian FIT-screening program of individuals aged 50 to 69 years in the Emilia-Romagna region assessed the effectiveness of adherence with invitation by comparing program attenders with nonattenders.^21^ With adjustments for self-selection, a 65% mortality reduction was observed in men (incidence-based CRC mortality rate ratio, 0.35; 95% CI, 0.29-0.41) and a 54% mortality reduction was observed in women (incidence-based CRC mortality rate ratio, 0.46; 95% CI, 0.37-0.58) after 11 years of follow-up.

A high participation rate is important to achieve a mortality reduction—only individuals who participate in screening can contribute to screen-detected cancers at an earlier, curable stage. Individuals who participate seem to continue, so the challenge is to get invitees to participate at least once.^22^ The participation rate in the early-start group (2008-2012) was approximately 60%, with a positivity rate of approximately 2% and a variable adherence with the follow-up colonoscopy by year, ranging from 86% to 92%.^12^ In an international comparison, the participation rate was generally high,^23^ and increased more than 10% when screening was changed to FIT in September 2015.^10^

There are significant challenges when measuring the effectiveness of ongoing screening programs in terms of reducing mortality. In this study, we avoided using a control group of individuals with CRC from a period before program implementation or invited nonparticipants. The Stockholm-Gotland screening program was initiated by randomly enrolling birth cohorts for comparison. Additionally, individuals with CRC diagnosed before the start of follow-up were excluded. Another strength of the study is its size and the high-quality individual data on screening history and follow-up for CRC diagnosis and cause of death. Almost 380 000 individuals were included in the study cohort. There were 14 years of follow-up data and 1723 CRC deaths. The results are strengthened by a second mortality measure, excess mortality, ie, independent of deciding the individual cause of death.

### Limitations

This study has some limitations. It is a difficult task to evaluate screening vs no screening program without biases in a population with an established screening. With 100% coverage of the CRC screening register, all screening invitations and participants in the program were registered, and approximately two-thirds of the individuals in the control group were invited to at least 1 of 5 screening rounds of the program 5 years or more after initiation of the program. This contamination of the control group hampers the evaluation of the full effectiveness of the screening program. Moreover, due to the long follow-up time to allow for death from CRC in the control group, more than 50% of the CRC deaths in the exposure group had their CRC diagnosis more than 2 years after the last screening invitation, ie, when the program’s preventive potential was low.

## Conclusions

This cohort study evaluating invitation to routine FOBT screening in Sweden found a 14% reduction in CRC mortality after 14 years of follow-up. Dilution of the estimated effectiveness is expected due to some later screening in the control group and some CRC deaths occurring among individuals who received a diagnosis years after invitations to screening had ended. Our results have an important public health implication in suggesting that organized population-based CRC screening with FOBT has the potential to save lives worldwide. By using FIT as screening test, with a higher sensitivity and participation rate than gFOBT, more lives could be saved, but adherence to prompt follow-up colonoscopies after a positive test result is essential.

## Notes

Supplement.
Data Sharing Statement
