# Supplementary material for: Routine Fecal Occult Blood Screening and Colorectal Cancer Mortality in Sweden
Source: JAMA Netw Open. 2024 Feb 27;7(2):e240516. doi: 10.1001/jamanetworkopen.2024.0516 (PMC10900849; doi:10.1001/jamanetworkopen.2024.0516)
Supplement: Supplement. — Data Sharing Statement [file jamanetwopen-e240516-s001.pdf]

Data Sharing Statement

Blom. Routine Fecal Occult Blood Screening and Colorectal Cancer Mortality in Sweden. *JAMA Netw Open*. Published February 27, 2024. doi:10.1001/jamanetworkopen.2024.0516

Data
Data available: No

Additional Information
Explanation for why data not available: The de-identified data are available for research upon request to Principal Investigator Johannes Blom and within the framework of the Swedish data protection legislation and any required permissions from authorities.
